# Is Phytomelatonin Complex Better Than Synthetic Melatonin? The Assessment of the Antiradical and Anti-Inflammatory Properties

**DOI:** 10.3390/molecules26196087

**Published:** 2021-10-08

**Authors:** Wirginia Kukula-Koch, Dominik Szwajgier, Katarzyna Gaweł-Bęben, Marcelina Strzępek-Gomółka, Kazimierz Głowniak, Henry O. Meissner

**Affiliations:** 1 Department of Pharmacognosy with Garden of Medicinal Plants, Medicinal University in Lublin, 1 Chodźki Str., 20-093 Lublin, Poland; 2 Department of Biotechnology, Microbiology and Human Nutrition, University of Life Sciences, 8 Skromna Str., 20-704 Lublin, Poland; dominik.szwajgier@up.lublin.pl; 3 Department of Cosmetology, University of Information Technology and Management in Rzeszów, 2 Sucharskiego Str., 35-225 Rzeszów, Poland; kagawel@wsiz.rzeszow.pl (K.G.-B.); mstrzepek@wsiz.rzeszow.pl (M.S.-G.); kglowniak@wsiz.rzeszow.pl (K.G.); 4 Therapeutic Research, TTD International Pty Ltd., 39 Leopard Ave., Gold Coast 4221, Australia; dr.meissner@ttdintnl.com.au

**Keywords:** synthetic melatonin, phytomelatonin complex, COX-2 inhibition, antiradical potential, DPPH, *Medicago sativa*, *Chlorella vulgaris*, *Oryza sativa*, HaCaT cells

## Abstract

This work aims to assess the recently established anti-inflammatory and antioxidant potential of melatonin of plant origin extracted from the plant matrix as a phytomelatonin complex (PHT-MLT), and compare its activity with synthetic melatonin (SNT-MLT) when used on its own or with vitamin C. For this purpose, a COX-2 enzyme inhibitory activity test, an antiradical activity in vitro and on cell lines assays, was performed on both PHT-MLT and SNT-MLT products. COX-2 inhibitory activity of PHT-MLT was found to be ca. 6.5 times stronger than that of SNT-MLT (43.3% and 6.7% enzyme inhibition, equivalent to the activity of acetylsalicylic acid in conc. 30.3 ± 0.2 and 12.0 ± 0.3 mg/mL, respectively). Higher antiradical potential and COX-2 inhibitory properties of PHT-MLT could be explained by the presence of additional naturally occurring constituents in alfalfa, chlorella, and rice, which were clearly visible on the HPLC-ESI-QTOF-MS fingerprint. The antiradical properties of PHT-MLT determined in the DPPH test (IC_50_ of 21.6 ± 1 mg of powder/mL) were found to originate from the presence of other metabolites in the 50% EtOH extract while SNT-MLT was found to be inactive under the applied testing conditions. However, the antioxidant studies on HaCaT keratinocytes stimulated with H_2_O_2_ revealed a noticeable activity in all samples. The presence of PHT-MLT (12.5, 25 and 50 µg/mL) and vitamin C (12.5, 25 and 50 µg/mL) in the H_2_O_2_-pretreated HaCaT keratinocytes protected the cells from generating reactive oxygen species. This observation confirms that MLT-containing samples affect the intracellular production of enzymes and neutralize the free radicals. Presented results indicated that MLT-containing products in combination with Vitamin C dosage are worth to be considered as a preventive alternative in the therapy of various diseases in the etiopathogenesis, of which radical and inflammatory mechanisms play an important role.

## 1. Introduction

Melatonin has been identified as “uncommonly effective” in reducing oxidative stress under a host of clinical circumstances [1]. This biogenic amine—a derivative of tryptamine (*N*-acetyl-5-methoxytryptamine)—was first isolated from the pineal gland of animals and later from humans [2,3]. It was identified as a hormone primarily responsible for regulating circadian rhythm and blood pressure. Numerous scientific publications confirm its multidirectional activity and effectiveness in the treatment of sleep disorders, insomnia, alleviation of shift work or jet lag symptoms, neoplastic diseases (tumors), microvascular ischemia or reperfusion injury, diabetes [4], and viral infections [5], including post-COVID syndrome [6]. According to the study of Loh and collaborators [7], the oral application of MLT in combination with vitamin C can be used for the prevention and combatting of viral infections in adult subjects. In another study, MLT [8] was proven to assist in the recovery of those infected through its anti-inflammatory properties. It indicates that even if MLT cannot directly eradicate the virus, this hormone prolongs patients’ survival time through its anti-inflammatory and antiradical potential, thereby ameliorating the immune system’s recovery and efficiently suppressing the virus.

Therapeutical properties of MLT are strictly related to its ability to increase the body’s own defense mechanisms that include a triggered release of cytokines and an induced synthesis of prostanoids and arachidonic and thromboxylic acids [9,10]. Nonetheless, the oxidative stress that is often present in several diseases, which disturbs the organisms’ homeostasis, can be overcome by the supplementation of MLT, which provides a prophylactic and therapeutic support [11,12].

As infection progresses, the increase in tryptophan production (in the indoleamine 2,3-dioxygenase/kynurenine metabolic pathway) causes a decrease in the MLT levels within the organism and induces a so-called “cytokine storm” [13]. MLT levels can also be lowered due to AhR activation via cytochrome P450 (CYP) 1B1 [14]. In light of the above- mentioned mechanism, the elevation of MLT in target body compartments can be one of the useful tools in the fight against several types of diseases. It is also conceivable that this elevation of MLT may counteract the disease-induced deleterious physiological effects on the human organism.

In 1995, MLT was detected in various organs of cultivated and wild plants [15,16,17]. According to Arnao [18] and Arnao and Hernandez-Ruiz [19], the MLT present in plants plays an important role in reducing the effect of stress on plants. Additionally, it acts as a growth stimulator, an antioxidant and pathogens repeller which works through specific melatonin receptors [18,19,20,21]. The melatonin of plant origin is characterized by the same chemical structure as the one detected in animals and in biochemical terms. Both are identical to the synthetic form of melatonin (SNT-MLT)—all inducing similar physiological responses in in vitro and in vivo models [13,14,22]. It is worth noting that the consumption of melatonin-rich plant-based food products and herbs can be treated as a major means of inducing physiologically effective melatonin. Stronger therapeutic effects of dietary MLT have been attributed to better bioavailability [4].

The above findings have led to the development of a report by Pérez-Llamas et al. [22], in which commercially manufactured therapeutic products based on a plant-origin complex containing melatonin (phytomelatonin complex) (PHT-MLT) have been marketed as dietary supplements in Spain, New Zealand, and the USA. It appears from the description of the three PHT-MLT products outlined by Pérez-Llamas et al. [22] that the New Zealand product is based on an extract from a single component: freeze-dried Montmorency tart cherry skin (*Prunus cerasus)*. The Spanish and the USA products represent a complexity of multicomponent extracts, derived from high melatonin-yielding dried plants/herbs, which are not disclosed by the Spanish manufacturer. The latter products are derived from the biomass of alfalfa (*Medicago sativa),* chlorella powder (*Chlorella vulgaris)*, and rice (*Oryza sativa)*. Another PHT-MLT product of Chinese origin [23] contains an extract from St John’s Wort herb [24].

Several recently reported works [1,7,25,26] suggest that synthetic melatonin and vitamin C, individually or together, may induce cell detoxifying action achieved via stimulation of antioxidative potential in physiological testing models, giving the base for a reasonable assumption that PHT-MLT could also be considered as a preventative measure and/or to combat both infectious diseases and diseases of civilization.

Considering the recently published research cited above, the present study was designed to explore the pharmacological potential of SNT-MLT and PHT-MLT preparation—alone and together, with an addition of vitamin C in terms of their anti-inflammatory and antioxidant properties. The multi-laboratory study was designed to find out to what degree the above assumptions may be supported by chemical fingerprinting, and measured antiradical potential (determined in in vitro spectrophotometric studies and on cell lines). The comparison of the analytically determined effectiveness of a pure form of synthetic melatonin and one of the preparations reported by Pérez-Llamas [22] MLT which contains melatonin and a complex of other metabolites extracted from the plant matrix of alfalfa, chlorella, and rice powders, both with or without vitamin C supplementation, was made. As well, an inhibitory activity of the above combinations towards cyclooxygenase-2 (COX-2) enzyme was assessed in a tailored assay.

## 2. Results

### 2.1. Qualitative and Quantitative Information on the Composition of MLT Samples Evaluated by the HPLC-ESI-QTOF-MS

**Figure 1:**
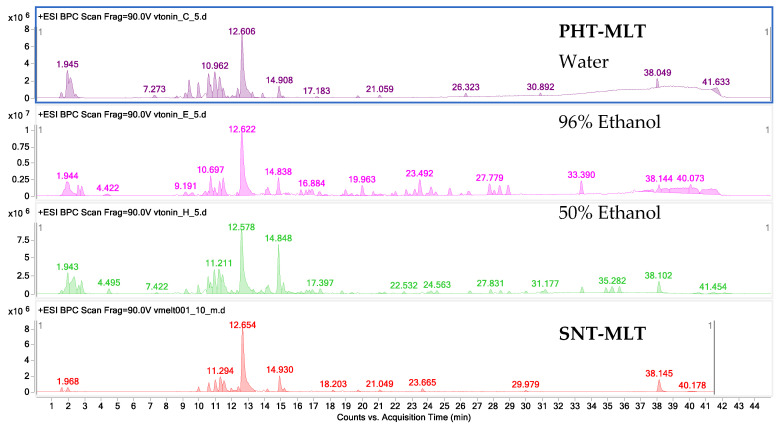
Mass chromatograms from the obtained PHT-MLT extracts and SNT-MLT (in the bottom) recorded in the positive ionization mode.

The applied chromatographic and spectrometric conditions led to a successful separation of metabolites which were present in the analyzed samples on the RP-18 column. In the performed analyses, the extracting solvents: water, 96% ethanol, and 50% ethanol were found to be important factors controlling the identity of the extracted metabolites from the plant matrix and their yield. The obtained fingerprints of the PHT-MLT extracts differ from one another in terms of both the intensity of similar signals and their quantity. The total ion chromatograms of all obtained extracts from the PHT-MLT powder presented together with the chromatogram of the SNT-MLT (Figure 1) in the positive ionization mode provide high sensitivity towards the detection of nitrogen-containing compounds with low molecular mass (50–1000 Da). MLT was identified in the PHT-MLT powder samples based on a direct comparison with the reference compound (SNT-MLT). The compound of interest was eluted after the 12th minute under applied analytical conditions. The recorded MS/MS spectra for both the SNT-MLT (A) and the one present in the PHT-MLT powder (B) presented in Figure S1 (Supplementary file) are identical, thus confirming the identity of melatonin.

Fingerprints of the tested extracts in the negative ionization mode showed that 96% and 50% ethanol (*v*/*v*) extracts from PHT-MLT powder had a higher content of secondary metabolites in comparison with its water extract (see Figure S2 in the Supplementary File). Under these conditions, the coexisting components were better recovered from the plant matrix. The negative ionization mode is commonly used to study the composition of phenolic compounds, and detected a rather complex composition of the two ethanol-based extracts in biochemical component terms, which may explain their higher antioxidant potential described in the following sections of the manuscript.

The quantitative data demonstrating the concentration of melatonin in the PHT-MLT samples were obtained based on the calibration curve drawn from the reference compound. Table 1 shows differences in MLT concentration depending on the extracting solvent used.

The quantitative data also shows that the addition of ethanol to the extraction protocol increases the recovery of this compound from the plant matrix. Interestingly, a similar extracting strength was noted for both 96% and 50% ethanol. The calculated content of MLT in the powder was used further in in vitro assays, thereby evaluating its biological activity. The antiradical and anti-inflammatory tests were aimed at comparing the properties of PHT-MLT extract containing 1.4% of MLT with the same concentration of SNT-MLT.

### 2.2. The Inhibition of COX-2 Enzyme by MLT-Containing Samples Alone and Together with the Solution of Ascorbic Acid

The inhibitory properties towards the COX-2 enzyme were assessed for PHT-MLT and SNT-MLT—alone and with the addition of 10 µL of ascorbic acid solution—as in the above-described antiradical tests. The obtained results indicate that the activity of PHT-MLT dried extracts (43.3%, equivalent to the activity of acetylsalicylic acid in conc. 30.3 ± 0.2 mg/mL) were some 5–6 times stronger than the activity of SNT-MLT (6.7% of COX-2 inhibition, equivalent to the activity of acetylsalicylic acid in conc. 12.0 ± 0.3 mg/mL), when measured at comparable concentrations of MLT in the tested samples. In the COX-2 assay, the addition of ascorbic acid solution to the tested PHT-MLT and SNT-MLT samples gave inconclusive results. In other words, the difference in the effect of vitamin C combined with MLT obtained from the two sources cannot be clearly distinguished. PHT-MLT dried extract combined with ascorbic acid inhibited 30.0 ± 0.0% COX-2 activity under the applied conditions (equivalent to the activity of acetylsalicylic acid in conc. 23.7 ± 0.1 mg/mL), whereas the percentage inhibition of SNT-MLT was calculated as 33.3 ± 0.0% (equivalent to the activity of acetylsalicylic acid in conc. 25.4 ± 0.0 mg/mL). Possible interpretation of the above outcomes may be that vitamin C could interfere with enzymatic reactions. This results in an observed color change of the tested samples suspended in the liquid medium during the incubation process.

### 2.3. The Assessment of the Antiradical Potential of Herbatonin, Melatonin, Ascorbic Acid and Their Combinations

DPPH scavenging activity test expressed as IC_50_ in mg of the PHT-MLT powder per mL was first performed on the dried total extracts obtained from PHT-MLT so as to compare their radical scavenging properties relative to the applied extraction conditions. Among the studied extracts the 50% ethanol solution was found to exhibit the strongest antiradical properties and was characterized by the lowest IC_50_ value of 21.6 ± 1 mg of PHT-MLT powder per mL (see Table S1 and Figure S2 in the Supplementary File).

This extract was proven to be ca. 200 times weaker than the tested ascorbic acid standard used in comparative tests. The weakest potential was obtained for water solutions produced from the powder. Conversely, the calculated IC_50_ value for the ascorbic acid solution (1 mg/mL) under the same testing conditions was equal to 0.12 mg/mL. Based on these results, the 50% ethanol extract of PHT-MLT was selected for further antiradical assessment and comparison with SNT-MLT and ascorbic acid solutions.

SNT-MLT samples analyzed at the same concentration as MLT from PHT-MLT powder did not reveal antiradical properties in this assay. Under the applied conditions, the tested volumes of SNT-MLT solution were found to be inactive and the determination of the IC_50_ value was not possible. None of the SNT-MLT samples, including all stock solutions, showed any detectable scavenging effects of DPPH radical (see Table S3 in the Supplementary File). It is reasonable to assume that the antioxidant potential of SNT-MLT reported in the scientific publications [27] could be the result of an impact of this compound on enzymes studied in in vivo models or in cell cultures.

Based on these results it could be concluded that the antiradical potential of PHT-MLT was related to the presence of other herbal components in the used plant species that are different from MLT itself, thereby supplementing its antioxidant combined impact on radicals in the model assay.

The following studies on the antioxidant potential of an ascorbic acid solution when applied together with SNT-MLT and PHT-MLT were performed to search for the eventual synergistic potential of the tested combinations of samples, by observing possible increase in the biological potency of resultant preparations.

**Figure 2:**
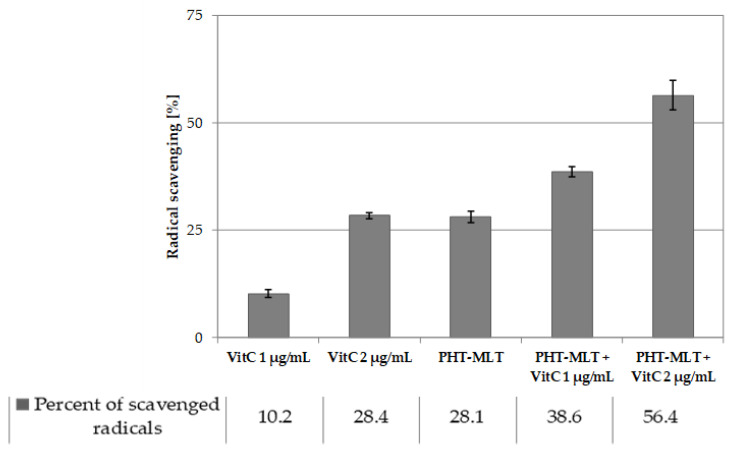
The percentage radical inhibition calculated for SNT-MLT and PHT-MLT alone and in combination with 1 and 2 µg/mL ascorbic acid solution (AsA); graph presents mean absorbance intensity for 30 min time point; the data on graphs show mean values ± SD that are representative for three experiments; *n* = 3.

Ascorbic acid has been already proven to exhibit strong antioxidant potential [28]. It has been treated as a reference compound in the antiradical assays to provide information on the relative strength of the tested samples. The solution of ascorbic acid investigated in this work at a concentration of 1 mg/mL showed the IC_50_ value of 0.12 mg/mL. Figure 2 (and Table S2 in the Supplementary File) summarizes the results obtained in tests when 10 and 20 µL (1 and 2 µg/mL) of vitamin C solution (1 mg/mL) were added to both SNT-MLT and PHT-MLT samples, with comparison made alongside the radical inhibition ratio of ascorbic acid alone.

The lowest volumes of ascorbic acid used in this study (10 and 20 µL) neutralized 10.2 ± 0.9% and 28.4 ± 0.8% of radicals present in the reactive solution.

The 50% ethanol extract of PHT-MLT (at the volume of 25 µL) with no addition of vitamin C scavenged 28.1 ± 1.4% of radicals (Figure 2). The addition of ascorbic acid (10 and 20 µL) led to an increase in the percentage inhibition of the tested mixture up to 38.6 ± 1.2% and 56.4 ± 3.4%, respectively. The obtained results indicate the existence of the cumulative antioxidant effect of PHT-MLT when supplemented with ascorbic acid.

However, the increase in the percentage inhibition value of both PHT-MLT and SNT-MLT solutions with 1 and 2 µg/mL of ascorbic acid represented an additive character of the relationship (see Figure 2 and Figures S2, S3 in the Supplementary File). The increase in their potency was equal to the percentage inhibition of ascorbic acid at the given volume. In this case, no synergistic effect was detected for the mixtures of herbatonin or synthetic melatonin when supplemented with ascorbic acid.

### 2.4. The Influence of MLT-Containing Samples and Ascorbic Acid on In Vitro ROS Generation of H_2_O_2_-Treated Keratinocytes

**Figure 3:**
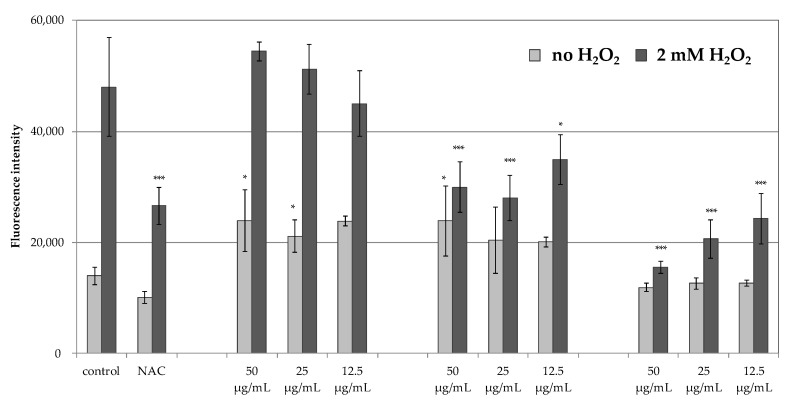
Mean fluorescence intensity values of H_2_DCFDA-loaded HaCaT keratinocytes treated with 50 µg/mL, 25 µg/mL or 12.5 µg/mL PHT-MLT, SNT-MLT, and vitamin C or 2 mM NAC as an antioxidant control, with or without the stimulation with 2 mM H_2_O_2_; increasing fluorescence intensity indicate high levels of intracellular ROS; graph presents mean fluorescence intensity for 180 min time point; the data on graphs show mean values ± SD that are representative for three experiments; *n = 4*, *** *p* < 0.001, * *p* < 0.05.

Results presented in Figure 3 shows that H_2_O_2_-treated HaCaT cells had an increased level of fluorescence, an indicator of intracellular ROS generation (dark bars), in comparison with untreated cells (light bars). Treatment of HaCaT cells with 50 µg/mL, 25 µg/mL or 12.5 µg/mL of dried PHT-MLT extract and vitamin C prior to H_2_O_2_ stimulation significantly reduced the production of intracellular ROS. The antioxidant effect of 50 µg/mL and 25 µg/mL dried PHT-MLT extract was comparable with 2 mM NAC, whereas vitamin C was a more potent antioxidant than 2 mM NAC of all tested concentrations.

## 3. Discussion

MLT [29] has been previously reported in several foods, including: corn (96.5 ng/g), wheat (124.7 ± 14.9 ng/g fresh weight—FW), grapes (8.9–158.9 ng/d dry weight—DW, tart cherries—Prunus cerasus cv. Balaton (13.46 ± 1.10 ng/g FW), pepper—Capsicum annuum cv. ‘F26′ (11.97 ng/g FW), tomato—Solanum lycopersicum cv. ‘Optima’ (14.77 ng/g FW and 249.90 ng/g DW), mustard seeds (black—189 ng/g DW and white—129 ng/g DW). Additionally, high quantities of MLT were found in dried mushrooms and nuts (12,900 ± 770 ng/g DW in Lactarius deliciosus mushrooms, and 233,000 ng/g DW in pistachios). Medicinal plants such as Hypericum perforatum and Scutellaria baicalensis were also assigned as sources of MLT (4490 and 7110 ng/g DW, respectively) [30].

Considering the above information, it is worth emphasizing that the tested preparation is composed of alfalfa (*Medicago sativa*), chlorella powder (*Chlorella vulgaris*), and rice (*Oryza sativa*) powders that are all included in the list of plants rich in MLT. Previous studies confirm the presence of this tryptamine derivative in rice at a varying level, depending on the cultivar also tested (from 0–264 ng/g DW) [31] in alfalfa powder at the level of 16 ng/g DW [19,32].

The fingerprint studies described in this article demonstrate the complexity of the PHT-MLT composition. Beside MLT itself, the PHT-MLT dried extracts contained several other phytoactive components such as chlorophyll, beta-carotene, isoflavones, phytates, saponins, and other plant constituents extracted from the plant material into a matrix product. The tentative identification of individual metabolites in the preparation is however difficult, as there are insufficient literature sources that could help to assign the recorded peaks. Other authors confirm the presence of primary metabolites, vitamins, and minerals in the extracts of used plant material, but the identity of low molecular weight secondary metabolites has not yet been sufficiently identified for these constituents individually, nor mixtures of them.

The research carried out by the authors in the initial stage of this laboratory study confirm that MLT is quickly extracted from the plant matrix into the water–ethanol environment. Ethanol and ethanol–water extracts were characterised by a higher content of this hormone as compared with water extracts, although, it is worth noting that acidified water extraction explored in the course of the laboratory extraction work, conducted in preparatory tests, showed encouraging results worthy of future practical considerations.

Our results confirm direct inhibitory activity of MLT towards the COX-2 enzyme. As far as the authors are aware, no similar effects of the direct inhibitory nature of MLT against COX-2 enzyme has been published. In the past, several tests showed that the commercially available MLT, similar to the one as used in this study, significantly attenuated the expression of COX-2 gene. However, no influence on the enzyme itself was reported [33]. The lack of similar reports can be explained by the fact that the test for the inhibitory activity of the COX-2 enzyme is not available for sale. In the presented study, we modified the method for the determination of the inhibitory activity towards the COX-2 enzyme. The Cayman enzyme assay kit is applied for the detection and quantification of the COX-2 enzyme activity in the animal tissues or after culturing the cell lines. We combined this COX-2 assay kit with the human recombinant COX-2 enzyme and thus modified a method to obtain a new, fast, and reliable assay for the direct measurement of COX-2 inhibitory properties in vitro. The adopted procedure is suitable for the determination of the inhibitory properties of COX-2 enzyme both by standard compounds and complex mixtures.

Previous publications mention some plant species and single metabolites with confirmed inhibitory properties against the COX-2 enzyme [34]. In the study of Cao and colleagues [35], several compounds were suspected of this activity. The most promising properties were assigned for resveratrol, senkyunolide O, and betulinic acid. Chandel and colleagues [36], in their review on COX-2 inhibitors of plant origin listed 3,3′,5-trihydroxy-2′-methoxybenzyl from Dioscorea opposita, 3-(2-ethyl-6-((3Z,7Z)-1,2,5, 6-tetrahydroazocin-5-yl) hexyl) morpholin-6-one—an alkaloid from Gracilaria opuntia, a coumarin—4-methylesculetin as well as other metabolites and their semi-synthetic derivatives that belong to the groups of phenolics and terpenes, as important drug candidates that act as anti-inflammatory agents.

The application of the COX-2 inhibition assay allowed us to confirm the anti-inflammatory potential of dried extracts from the PHT-MLT product and SNT-MLT samples used in this study which exhibited a 5–6 times stronger activity of the phytomelatonin complex, possibly die to the presence of other natural products.

Additionally, the antioxidant potential of PHT-MLT dried extracts and SNT-MLT samples studied in in vitro spectrophotometric tests differed significantly, with PHT-MLT inducing neutralization of the DPPH radical to a much higher extent than the SNT-MLT. Weak activity and high IC_50_ values calculated for SNT-MLT suggested that the broadly described potential of N-acetyl-5-methoxytryptamine could be due to its interaction with ROS-scavenging enzyme systems and not due to the neutralization of produced radicals. The above conclusions are in accordance with reported by Yu and co-investigators [37], of the ability of MLT inducing reactions characteristic of superoxide dismutase (SOD), glutathione, glutathione peroxidase, ascorbate peroxidase, catalase, or other enzymes. According to the above authors, MLT is capable of upregulating the levels of these enzymes by altering gene expressions, and by this activity, induce the antioxidant potential in a living cell.

At the same time, the performed in this work in vitro tests with the DPPH radical obtained in this work, underline a marked potential in a direct radical scavenging action of other constituents present in the powder matrix of PHT-MLT extract. A 50% ethanol PHT-MLT dried extract was found to exhibit the strongest antiradical potential with the IC_50_ value calculated as 21.6 ± 1 mg of powder/mL. This particular extracting solvent has been previously selected by other authors as the most effective in the recovery of antioxidants from the plant matrix [38]. Moreover, under the experimental conditions applied in this study, calculation of the IC_50_ value of MLT standard was not possible, which confirms low antiradical potential of the solution. The antiradical activity assessment that was carried out for both PHT-MLT and SNT-MLT in conjunction with vitamin C allowed observation of the additive action mechanism between the two types of compared samples. The calculated IC_50_ values of the mixture of PHT-MLT dried extract and vitamin C decreased exactly by the value measured for the vitamin C solution alone. This demonstrates the cumulative/additive antiradical potential of PHT-MLT used in conjunction with the vitamin C. The same conclusions were drawn when comparing the antiradical potential of MLT standard with ascorbic acid solution (see Tables S2 and S3 in the Supplementary file). It is worth adding, that the other occurring in the PHT-MLT sample natural products did not affect the extract on the antiradical potential of ascorbic acid.

The inability to obtain satisfactory antiradical results for the MLT encouraged the authors to assess the antioxidant potential of PHT-MLT in comparison with SNT-MLT and vitamin C, using an in vitro experimental model in which the generation of ROS was measured intracellularly in human immortalized keratinocytes HaCaT. The cells were induced with 2 mM H_2_O_2_ and the production of ROS was measured using H_2_DCFDA fluorogenic dye. The reduction in intracellular ROS levels was observed for PHT-MLT dried extract and vitamin C treatment, whereas SNT-MLT did not influence the intracellular ROS generation. The influence of SNT-MLT on intracellular ROS in HaCaT cells was previously analyzed by Lee and co-workers [38]. In this study the treatment of the cells with 300 µM H_2_O_2_ in combination with 1 mM (232.28 µg/mL) or 2 mM (464.56 µg/mL) SNT-MLT decreased intracellular ROS by ca. 16% in comparison with H_2_O_2_-treated cells. In another study by Park et al. [39] SNT-MLT at 1 mM and 2 mM was shown to reduce intracellular ROS levels in tert-butyl hydroperoxide (t-BOOH) treated HaCaT keratinocytes [39]. The lack of antioxidant effect in SNT-MLT-treated cells in this study could be explained by low concentrations of melatonin (50, 25 and 12.5 µg/mL corresponding to 215, 107.5, and 53.75 µM, respectively). Significantly lower levels of intracellular ROS generated in the presence of PHT-MLT suggest that the plant-derived melatonin is effective in lower concentrations than SNT-MLT.

In addition to H_2_O_2_-treated keratinocytes, the intracellular ROS levels were also measured in the cells without this treatment, grown in the presence of 50, 25, or 12.5 g/mL SNT-MLT, PHT-MLT, and vitamin C alone. In these experiments, both SNT-MLT and PHT-MLT dried extract slightly increased intracellular ROS levels. The effect of SNT-MLT alone on ROS production in keratinocytes was not shown in the previously mentioned studies by Lee [39] and Park [40]. However, increased intracellular ROS was previously described by McConnell and co-workers in SNT-MLT-treated glioblastoma cell lines U87-MG and MU1454. SNT-MLT also increased activation of glutathione peroxidase, one of the major antioxidant enzymes [40]. These seemingly contradictory observations were recently explained by Gonzales et al. [41] on pancreatic stellate cells (PSC). In this study SNT-MLT (1 mM, 100 µM, 10 µM, or 1 µM) treatment of PSC-elevated intracellular ROS production caused depolarization of the mitochondrial membrane potential, and decreased the GSH/GSSG ratio and a concentration-dependent increase in the expression of the antioxidant enzymes. Based on the obtained data, Gonzales and co-workers assumed that the prooxidant conditions evoked by SNT-MLT might activate the intracellular signaling pathways in an attempt to counteract the pro-oxidative state. SNT-MLT was shown to upregulate the expression of the nuclear factor erythroid 2–related factor 2 (Nrf2), an emerging regulator of cellular resistance to oxidants. Nfr2 activates the antioxidant response element (Keap1-ARE) pathway, leading to increased expression of antioxidant enzymes including superoxide dismutase (SOD), glutathione peroxidase (GP), hemoxygenase 1 (HO-1), and NADPH: quinone oxidoreductase (NQO1). SNT-MLT activates this pathway to induce protective antioxidant actions [42,43,44]. As the increase in intracellular ROS levels are observed also in response to PHT-MLT treatment it is possible that that the antioxidant activity of PHT-MLT is based on the same principle.

The results obtained in the current in vitro studies confirm that PHT-MLT has an important impact on the antioxidant potential of cells. Its activity may be related more to the direct effect of increasing the synthesis of intracellular enzymes responsible for free radicals scavenging than to the effect of neutralizing free radicals.

As previously mentioned, the antioxidant action of SNT-MLT was confirmed in the in vitro tests by Lee and co-investigators [38]. They observed a 16% reduction in the intracellular ROS value at the concentration of 1 mM (or approx. 232.28 µg/mL) of SNT-MLT. In the present study, the PHT-MLT dried extract was five to ten times more active than SNT-MLT (as judged using ROS methods and PHT-MLT concentrations as low as 50 µg/mL or 25 µg/mL, respectively). This trend was consistent with the result obtained in the COX-2 assay where PHT-MLT resulted in five to six times higher activity than SNT-MLT.

Based on the above findings it is reasonable to assume that the dietary supplementation of PHT-MLT, alone or in combination with vitamin C and/or other phytonutrients such as quercetin or curcumin, may provide prophylactic and/or adjuvant therapy against infectious and civilization diseases by helping the organism to regain its homeostasis. This could be supported by the findings reported in the study of Loh [7], who strongly confirmed the above assumptions that oral application of MLT in combination with vitamin C could be used for both preventing and combating COVID-19 in infected adult subjects. It has also been established that oxidative stress plays a role in the pathogenesis of COVID-19 by perpetuating the cytokine storm cycle, blood clotting mechanism, and exacerbates hypoxia [45]. Based on these observations, it could be concluded that the supplementation of a diet with various pure compounds, displaying antioxidant activity, may help to boost the immune system and provide prophylactic and therapeutic support, not only against COVID-19 infections specifically, but also against other related or unrelated viral infections [11,12].

Additionally, in areas of infection and those health conditions with elevated levels of free radicals the COX-2 enzyme is activated under the influence of factors related to inflammation and catalyzes the reaction of prostanoids, thromboxylic, and arachidonic acid syntheses. In consequence, the induced “cytokine storm” appears to be caused by an activation of indoleamine 2,3-dioxygenase/kynurenine route. The final result is the decreased response of the organism. This topic has been lately comprehensively discussed in several works, e.g., in an excellent review by Anderson and colleagues [13]. To counteract this type of physiological disturbance, the administration of immune-boosting supplements suppressing the ongoing oxidative stress, acute-inflammation, and cytokine storm should be applied so that destruction and damage caused to affected tissues is prevented [46].

As the above-presented results show, PHT-MLT is certainly the preparation capable of reducing the effects of physiological stress in the body and it deserves further attention due to its proven COX-2 inhibitory properties. These features are important when considering the treatment of viral diseases or when preventing the development of civilization diseases whose ethiopathogenesis is often based on the activity of radicals [12].

Despite the fact that *N*-acetyl-5-methoxytryptamine is obtained synthetically or from animal glands, the last decades have brought a lot of data confirming its presence in raw plant material. As mentioned above, PHT-MLT which occurs in plants in a rich mixture of bioactive metabolites of different biochemical origin, in comparison with SNT-MLT, may be characterized by a better absorption and availability in the human organism and by an extended metabolic action. Apparently, the accompanying concentrated ingredients embedded in the matrix of the extracted plant material used in this study may be one of the contributing factors responsible for influencing stability and observed improved pharmacokinetic profile of the tested PHT-MLT.

Extrapolating results from ROS and COX-2 assays presented in the study led to the suggestion that protective PHT-MLT doses which may prevent and counteract viral infections, could be as high as 5.0 mg to 50 mg per day for symptomatic adult human subjects, as suggested by Loh [7]. For asymptomatic subjects and for the prevention of civilization diseases, applying a dose between 0.2 mg to 0.5 mg PHT-MLT per day together with 1 g per hr of vit. C (10 g to max 18 g) can be of assistance [7].

Results from work on PHT-MLT presented in this paper and supported by other previously cited authors provide further interpretation of hypothetic therapeutic action of PHT-MLT when combined with vitamin C in protection against non-specific viral infections, civilization diseases including cancers, atherosclerosis, dementia, diabetes, autoimmune disorders, and others.

## 4. Materials and Methods

### 4.1. Material

Commercial sample of 500g PHT-MLT used in this work was represented by “*Herbatonin^®^* Powder” obtained from the bulk batch of the marketed product (DHBT-12-3432) manufactured by Natural Health International Co. (NHI-USA), 2550 S Decker Lake Blvd Ste 28, West Valley City, UT 84119, USA, which formulates the bulk product into capsule form containing either 300 µg or 3 mg of plant-based melatonin (melatonin-equivalent) for international phytopharmaceutical and dietary supplement markets. Tested proprietary product *Herbatonin^®^* Powder has been declared to contain undisclosed proportions of aqueous extract from *Medicago sativa* harvested in vegetative, before flowering stage, *Chlorella vulgaris* and pulverized *Oryza sativa* (although in earlier years *Chlorella pyrenoidosa* was used prior to exporting the product to the EU countries where according to the current EU regulations *Chlorella vulgaris* is accepted only).

The commercially available SNT-MLT (5 mg tablets) was obtained from Lekam company. The standard of vitamin C (L-(+)-ascorbic acid) and MLT at the purity of 95% was obtained from Sigma Aldrich (St. Louis, MO, USA).

### 4.2. Extraction and Sample Preparation

0.5 g of PHT-MLT powder was placed in Eppendorff 15 mL tubes and sonicated for 30 min at the room temperature in 5 mL of extracting solvent: water, 96% ethanol (*v/v*) or 50% ethanol (*v/v*). The solid to liquid ratio was selected in the initial studies. All extracts were prepared in triplicate—two on one day and the remaining on the next day. All extracts were evaporated under vacuum and at 45 °C in the rotary evaporator and were later rediluted in the suitable solvents for both compositional and biological activity tests. All dried residues were stored at −20 °C before the experiments.

For SNT-MLT: 5 mg tablets of melatonin were crushed in a mortar and dissolved in the same solvents as PHT-MLT to the volume that resembled the concentration of MLT in PHT-MLT samples. The stock solution of vitamin C (10 mg/mL) was prepared with an acidified water (pH = 3.2) immediately before to the experiments. All samples were filtered through a nylon syringe filter (nominal pore size of 0.22 µm) (Merck, Darmstadt, Germany) and subjected directly to the HPLC-MS analysis or diluted to expected concentrations for the antiradical assays.

### 4.3. Reagents

Standards of gallic acid, L-(+)-ascorbic acid, melatonin, K_2_S_2_O_8_, 2,2′-Azino-bis(3-ethylbenzothiazoline-6-sulfonic acid) diammonium salt (ABTS) and 2,2-diphenyl-2-picrylhydrazyl (DPPH) at the purity exceeding 95% were purchased from Sigma Aldrich (St. Louis, MO, USA). Reagent grade methanol, ethanol, DMSO, hydrochloric acid, and ammonia were obtained from Avantor Performance Materials (Gliwice, Poland). The HPLC-MS reagents: formic acid, acetonitrile and water of HPLC-MS grade were produced by Merck (Darmstadt, Germany). The reagents for the antioxidant activity determination in cells: immortalized human keratinocytes HaCaT were purchased from CLS Cell Lines Service GmbH (Eppelheim, Germany), fetal bovine serum (FBS) was obtained from Pan-Biotech (Aidenbach, Germany), Dulbecco’s modified Eagle’s medium (DMEM)/high glucose, with and without phenol red, Dulbecco’s phosphate-buffered saline (DPBS), 2′,7′-dichlorofluorescin diacetate (H2DCFDA) and N-acetylcysteine (NAC) were purchased from Sigma Aldrich (St. Louis, MO, USA). For the COX-2 inhibition assay, reagents from Cayman COX Activity Assay Kit (No. 760151) were used together with the COX-2 enzyme (Human recombinant, Cayman No. 60122).

### 4.4. The HPLC-ESI-Q-TOF-MS-Based Study on the Composition and Quantity of MLT in the Tested Samples

An HPLC-ESI-Q-TOF-MS-based qualitative and quantitative analysis of PHT-MLT extracts and melatonin standard was achieved in a tailored method run on an Agilent G3250AA LC/MSD Q-TOF system equipped with an HP 1200 chromatograph and an ESI- Q-TOF-MS spectrometer (Agilent Technologies, Santa Clara, CA, USA). The set was composed of a degasser (G1322A), a thermostated column chamber, an autosampler (G1329B), a PDA detector (G1315D), and a binary pump (G1312C). The analyses were performed in a gradient method on a Zorbax RP 18 (150 × 2.1 mm, dp = 3.5 µm) HPLC column. The composition of gradient and the selected mass spectrometer settings were presented in Table 2.

The quantitative evaluation of the melatonin was performed based on the calibration curve prepared out of 5 solutions by dissolving of the stock solution of 1 mg/mL to 0.02, 0.01, 0.005, 0.0025 and 0.00125 mg/mL to include the range of content of the individual metabolites in the extracts. The R2 value of all of them exceeded 0.998 and the following calibration curve equation was obtained: y = 10201555384 x − 407111.

For the MS/MS spectra, a data-dependent method was constructed which enabled the fragmentation of the two biggest detected peaks in each microscan. After the collection of one spectrum, these two peaks were ignored from fragmentation for the following 0.3 min.

### 4.5. The Antiradical Properties of Herbatonin, Melatonin and Vitamin C in the 1,1-Diphenyl-2-Picrylhydrazyl (DPPH) Assay

The antiradical properties evaluated in the DPPH test were performed on the PHT-MLT extract, SNT-MLT, and ascorbic acid solutions. The samples’ concentrations were selected based on the results obtained from the quantitative analyses on HPLC-MS to compare with each other the antiradical effects of equivalent concentrations of melatonin in both PHT-MLT and SNT-MLT samples. The assay was conducted according to the previously published procedure [47].

For the study, a 0.3 mM ethanol solution of DPPH radical was prepared immediately before the analysis. Its absorbance was first checked at 515 nm by a Unicam Helios Gamma spectrophotometer (Thermo Electron Corporation) and was adjusted with ethanol not exceeding 1. The solution was kept in the refrigerator in between runs and was used within one working day. In the assay, 0.2 mL of a tested sample was mixed with 1.8 mL of DPPH radical solution in a reaction vial and was kept in the darkness for 30 min at 37 °C. The absorbance of the solutions was measured at 515 nm. All measurements were performed at least three times and in two separate series performed on two consecutive days. The obtained results were calculated relative to the blank that was prepared from 0.2 mL of DMSO and 1.8 mL of DPPH solution. Ethanol was used as a negative control. To calculate the IC_50_ values, five solutions were tested for each herbatonin extract (0.5 g of PHT-MLT powder for 5 mL of a solvent) and melatonin solution (0.14 mg/mL). For both samples 200, 100, 50, 25 and 12.5 µL of stock solutions were used in the assay and where necessary the volume was supplemented to 200 µL with DMSO. For the solution of ascorbic acid (0.2 mg/mL) the following volumes were tested: 100, 80, 60, 40, 20, and 10 µL. They were also diluted to 200 µL with DMSO prior to the experiment.

The percentage inhibition of radicals’ formation was calculated based on the following formula:

Inhibition percent [%] = [(ΔA_0_ − ΔA_A_)/ΔA_0_] · 100% (1)

where A_0_ was the absorbance of the solution after 30 min in the presence of the tested solutions, and A_A_ was the absorbance of the blank.

The calculated IC_50_ values showed the concentration of a sample that was required to scavenge 50% of DPPH free radicals. The determination of IC_50_ values was the ground of scavenging activity comparison between the studied extracts of PHT-MLT obtained in different conditions and with the corresponding concentration of SNT-MLT. Additionally, the antiradical activity assessment was performed for the tested samples with and without the addition of ascorbic acid to discuss the possible occurrence of interactions.

### 4.6. The Antiradical Properties of Herbatonin, Melatonin and Vitamin C in the 1,1-Diphenyl-2-Picrylhydrazyl (DPPH) Assay

For the assay, reagents from Cayman COX Activity Assay Kit (No. 760151) were prepared as suggested by the producer and combined with COX-2 enzyme (Human recombinant, Cayman No. 60122, pre- diluted 100-fold using 100 mM, pH 8.0 Tris buffer). An appropriate volume of each studied sample (see Table 3) corresponding to the performed in vitro antioxidant studies was mixed with 10 µL of hemin, shaken and left for 5 min at 25 °C followed by the addition of 20 µL colorimetric substrate, arachidonic acid solution and completed to 18 µL by Tris buffer (100 mM, pH 8.0). To start the reaction, 0.02 mL of COX-2 enzyme solution was added.

The COX-2 inhibitory potential of PHT-MLT and SNT-MLT was studied also in combination with ascorbic acid water solution (0.2 mg/mL) so as to test for the existence of potential synergistic reactions between the MLT-containing samples and vitamin C. In this case, a volume of 10 µL of ascorbic acid solution replaced the same volume of the Tris buffer in the reaction mixture.

An increase of the absorbance during the incubation at room temperature was recorded at 590 nm (Tecan microplate reader, Grödig, Austria) after 20 min. Negative (blank) sample (10 µL mL Tris buffer instead of the studied sample) and positive control (COX-2 inhibitor DuP-697 to check the method) were run simultaneously. Additionally, samples containing corresponding volumes of DMSO or ethanol were run at the same time to check the effect of DMSO and alcohol on the enzyme activity. Background of the studied samples (10 µL of the sample mixed with 19 µL buffer) was also measured and included in the calculations. Each sample was run in at least 4 repeats.

Inhibition of the enzyme activity was expressed in % (indicates by how many % the activity has been reduced in relation to the negative (blank) sample for which the maximum activity was assumed as 100%, under the conditions used in the method). Additionally, inhibition of enzyme activity was expressed as acetylsalicylic acid equivalent concentration (mg/cm^3^). For this purpose, acetylsalicylic acid solutions (Sigma-Aldrich, St. Louis, MO, USA, A5376, >99%) were prepared at 14 concentrations (0.2–10 mg/cm^3^). Acetylsalicylic acid solutions were analyzed similarly to tested samples.

### 4.7. The Antiradical Properties in HaCaT Cells

The influence of PHT-MLT, SNT-MLT, and ascorbic acid on the generation of intracellular levels of reactive oxygen species (ROS) was analyzed in human immortalized keratinocytes HaCaT using 2′,7′-dichlorofluorescin diacetate (H_2_DCFDA) assay, as described by Wu and Yotnda [48]. After diffusion into the cell, H_2_DCFDA is deacetylated by cellular esterases and subsequently oxidized by ROS into 2′,7′-dichlorofluorescein (DCF). The increase of the DCF fluorescence over time indicate elevated intracellular ROS levels [49]. HaCaT cell line was maintained in DMEM supplemented with 10% FBS at 37 °C in a humidified atmosphere with 5% CO_2_. For the experimental purposes, 1 × 10^4^ HaCaT keratinocytes were plated per well onto black-walled, 96-well plates (Biokom, Janki, Poland) and cultured overnight in DMEM supplemented with 10% FBS. The cells were loaded with 5 µM H_2_DCFDA diluted in serum-free, phenol red-free DMEM at 37 °C and 5% CO_2_ for 30 min, in darkness. Diluted compounds (final concentration 50 µg/mL, 25 µg/mL and 12.5 µg/mL) or a known ROS-scavenger *N*-acetyl-l-cysteine (NAC, 2 mM) were pre-mixed in serum-free, phenol red-free DMEM with 2 mM H_2_O_2_ and applied to H_2_DCFDA-loaded cells. Equal volume of the serum-free, phenol-red free DMEM was applied to the control cells. The cells were then incubated at 37 °C and 5% CO_2_ in darkness. The fluorescence intensity of the forming 2′,7′-dichlorofluorescein (DCF) was measured following 30, 60, 120, 180, and 240 min of incubation using FilterMax F5 microplate reader (Molecular Devices, San Jose, CA, USA) at maximum excitation and emission spectra of 485 and 535 nm, respectively.

### 4.8. Satistical Analysis

All experiments were conducted in at least three replicates. The obtained data were analyzed using Microsoft Office Excel Software 2010 and GraphPad Prism 7.0 Software (GraphPad Software, San Diego, CA, USA), unless otherwise specified above. The statistical significance between results obtained for in vitro antioxidant activity was analyzed using one-way ANOVA followed by Tukey’s test.

## 5. Conclusions

The results presented in this work on the phytomelatonin preparation containing plant-based melatonin in a complex of three blended herbal components, exhibits its ability to alleviate intracellular free radicals’ levels and to reduce inflammatory conditions in the laboratory models under a wide range of in vitro assay conditions.

The PHT-MLT powder that is composed of *Medicago sativa*, *Chlorella vulgaris*, and *Oryza sativa* species revealed stronger COX-2 inhibition (ca. 6.5 times stronger) and higher antiradical potential from synthetic melatonin that was demonstrated by the results obtained in in vitro assays, as applied in this study. However, when tested directly on HaCaT keratinocytes, stimulated with H_2_O_2_, the antiradical potential value measures for PHT-MLT and SNT-MLT were noticeably decreased, thereby indicating the lowering effect of antioxidative stress of tested cells used in the assays.

The antioxidant properties of melatonin-containing samples tested on its own and in combination with Vitamin C are worth considering for use in alleviating symptoms of infections and preventing the progression of civilization diseases, with the assumption that it may help patients to decrease adverse effects of free radical activity in the human body. Taking into consideration the antioxidant properties and COX-2 inhibitory properties from the SNT- MLT, a richer chemical profile of the PHT-MLT preparation in comparison with SNT-MLT, together with a previously improved intestinal absorption of MLT from natural resources, makes it more efficient concerning the antioxidant properties and COX-2 inhibitory properties from the SNT-MLT solution.

## Figures and Tables

**Table 1:** The content of melatonin in the extracts from PHT-MLT powder calculated from the corresponding mass chromatograms (SD—standard deviation).

Type of Extract	Percentage Content of Melatonin in the Powder [%]	SD
Water	1.171	0.04
96% Ethanol	1.417	0.15
50% Ethanol	1.418	0.14

**Table 2:** The chromatographic parameters set for the qualitative analysis of the tested samples of PHT-MLT powder and SNT-MLT (*ACN—acetonitrile, FA—formic acid*).

Parameter	Value		
Gradient of solvents	Time	ACN + 0.1%FA	H_2_O + 0.1%FA
	0 min	2	98
	2 min	10	90
	6 min	40	60
	35–36 min	95	5
	37 min	2	98
The total length of the run	45 min + 2 min postrun		
Injection volume	5 µL		
Flow rate	0.2 mL/min		
HPLC thermostat temperature	25 °C		
Mass detector settings:			
- *m/z* range. - Scan speed. - Fragmentation energy. - Collision energies. - Gas and sheath gas flows. - Gas and sheath gas temp. - Nebulizer pressure. - Capillary voltage.	50–1200 u 1 scan/second 90 V 10 and 20 V 12 L/min 325 and 300 °C 35 psig 2500 V		
Data handling	MassHunter Qualitative Analysis Navigator version: B.08.00		

**Table 3:** The experimental design and volumes of the tested samples, with and without the addition of vitamin C (AsA) applied in the study on COX-2 inhibitory properties.

Sample.	Volume(s) of Tested Samples in the Reaction Mixture:
PHT-MLT (1)	30 µL of PHT-MLT 50% ethanol extract in DMSO
SNT-MLT (3)	30 µL of SNT-MLT
1 + AsA	30 µL of PHT-MLT 50% ethanol extract in DMSO + 10 µL ascorbic acid solution
3 + AsA	30 µL of SNT-MLT + 10 µL of ascorbic acid

## Data Availability

The supporting information can be found in the Supplementary Materials.

## Supplementary Materials

Figure S1: The comparison of the MS/MS spectra of melatonin recorded for the phytomelatonin powder and synthetic melatonin at the collision energies of 10 V. Figure S2: The total ion chromatograms recorded for the obtained extracts of PHT-MLT powder in the negative ionization mode. Figure S3: The relationship between the antiradical properties of 50% ethanol extract from PHT-MLT powder expressed in percent of inhibition and quantity of the powder in miligrams compiled to calculate the IC50 value of the extract. Table S1: The antiradical potential calculated for three types of extracts obtained from herbatonin powder expressed as IC50 values. Table S2: The scavenging properties (% inhibition of radicals) obtained for the ascorbic acid stock solution (0.2 mg/mL). Table S3: The percentage of scavenged DPPH radicals calculated for SNT-MLT (5 mg/mL) alone and in combination with vitamin C solution (0.2 mg/mL).
