# Supplementary material for: Is Phytomelatonin Complex Better Than Synthetic Melatonin? The Assessment of the Antiradical and Anti-Inflammatory Properties
Source: Molecules. 2021 Oct 8;26(19):6087. doi: 10.3390/molecules26196087 (PMC8512846; doi:10.3390/molecules26196087)
Supplement: Supplementary file 1 [file molecules-26-06087-s001.zip › molecules-1393915-supplementary.pdf]

SUPPLEMENTARY FILE

Is Phytomelatonin Complex Better Than Synthetic Melatonin? The Assessment of the Antiradical and Anti-Inflammatory Properties

Wirginia Kukula-Koch [1,*], Dominik Szwajgier [2], Katarzyna Gaweł-Bęben [3], Marcelina Strzępek-Gomółka [3], Kazimierz Głowniak [3] and Henry O. Meissner [4]

1 Department of Pharmacognosy with Garden of Medicinal Plants, Medicinal University in Lublin, 1, Chodźki str., 20-093 Lublin, Poland

2 Department of Biotechnology, Microbiology and Human Nutrition, University of Life Sciences, 8, Skromna str. 8, 20-704 Lublin, Poland; dominik.szwajgier@up.lublin.pl

3 Department of Cosmetology, University of Information Technology and Management in Rzeszów, 2, Sucharskiego str., 35-225 Rzeszów, Poland; kagawel@wsiz.rzeszow.pl (K.G.-B.); mstrzepek@wsiz.rzeszow.pl (M.S.-G.); kglowniak@wsiz.rzeszow.pl (K.G.)

4 Therapeutic Research, TTD International Pty Ltd., 39 Leopard Ave., Elanora, QLD 4221, Australia; dr.meissner@ttdintnl.com.au

* Correspondence: virginia.kukula@gmail.com

Figure S1. The comparison of the MS/MS spectra of melatonin recorded for the phytomelatonin powder and synthetic melatonin at the collision energies of 10 V.

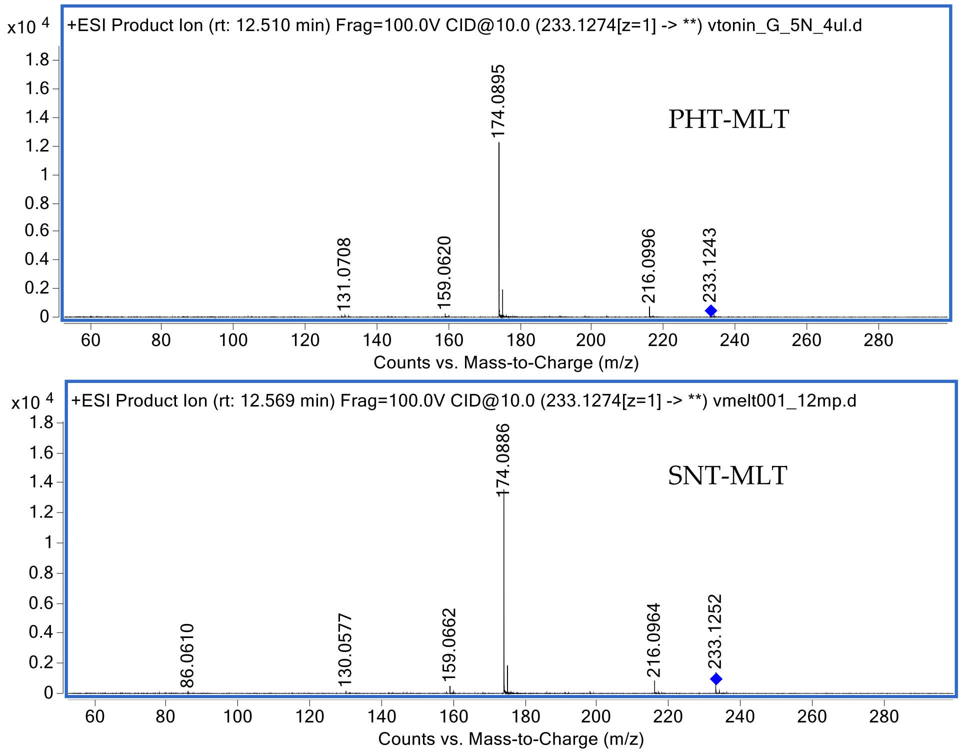

Figure S2. The total ion chromatograms recorded for the obtained extracts of PHT-MLT powder in the negative ionization mode.

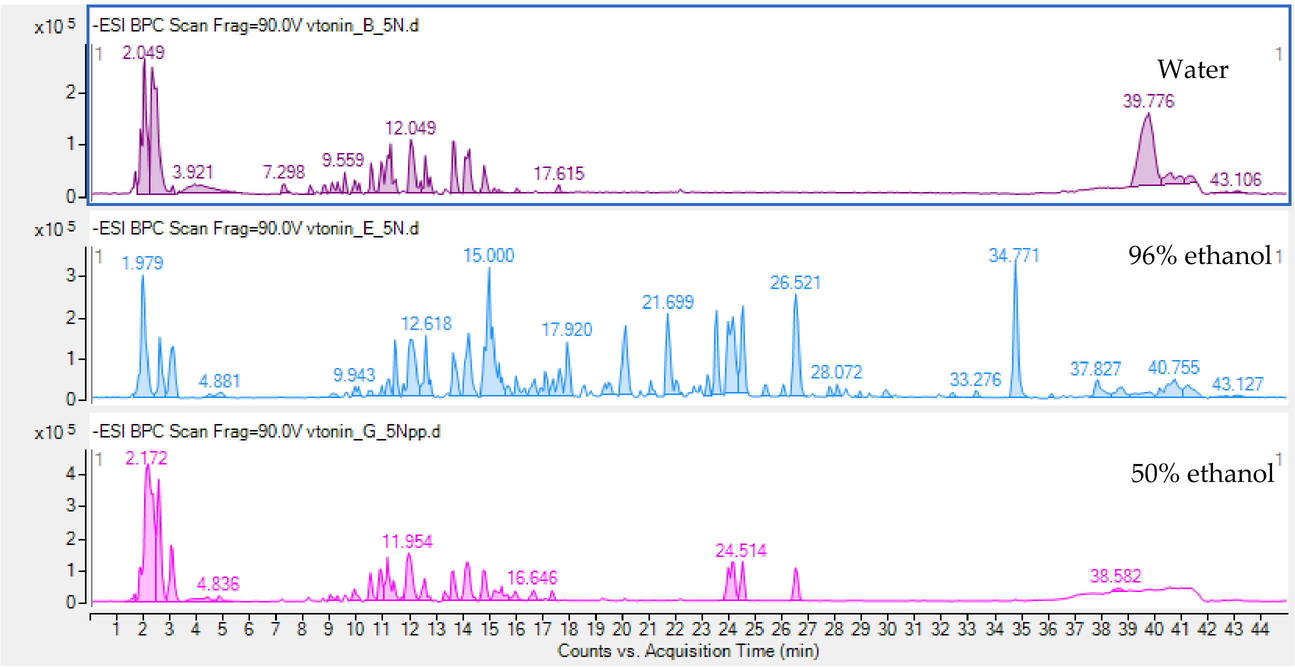

Table S1. The antiradical potential calculated for three types of extracts obtained from herbatonin powder expressed as IC_{50} values

Extract	IC_{50} value [mg of powder/mL]	Standard deviation	Initial concentration
Water	78.7	5.5	
96% Ethanol	61	4	1 g/ 10 mL
50% Ethanol	21.7	1	
**Ascorbic acid**	**0.12 mg/mL**		1 mg/mL

Figure S3. The relationship between the antiradical properties of 50% ethanol extract from PHT-MLT powder expressed in percent of inhibition and quantity of the powder in miligrams compiled to calculate the IC50 value of the extract.

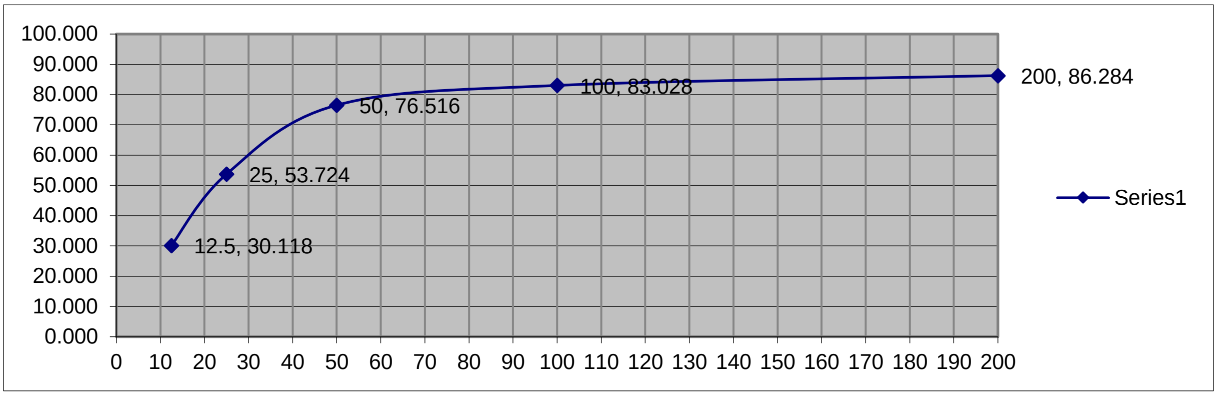

Table S2. The scavenging properties (% inhibition of radicals) obtained for the ascorbic acid stock solution (0.2 mg/mL)

The volume collected from the stock solution [µL]	Absorbance value obtained	% of scavenged DPPH radicals
**100**	0.052	95
**80**	0.101	90.3
**60**	0.148	85.8
**40**	0.181	82.63
**20**	0.750	28.02
**10**	0.932	10.55

Table S3. The percentage of scavenged DPPH radicals calculated for SNT-MLT (5 mg/mL) alone and in combination with vitamin C solution (0.2 mg/mL)

Synthetic melatonin / sample volume [µL]	SNT-MLT	SNT-MLT + 10 µL vitamin C	SNT-MLT + 20 µL vitamin C
**200**	17.384	32.679	43.861
**100**	17.384	30.886	44.072
**50**	18.439	30.886	43.650
**25**	17.384	31.941	43.966
**IC50 [mg /mL]**	-		
